# GDF15 knockdown suppresses cervical cancer cell migration *in vitro* through the TGF‐β/Smad2/3/Snail1 pathway

**DOI:** 10.1002/2211-5463.13013

**Published:** 2020-11-08

**Authors:** Li Li, Ruihong Zhang, Hailei Yang, Donghua Zhang, Jiwei Liu, Jinfang Li, Bin Guo

**Affiliations:** ^1^ Department of Gynaecology Binzhou Medical University Hospital Binzhou China; ^2^ Department of Obstetrics Chengwu People's Hospital Heze China; ^3^ Department of Gynaecology The Liaocheng Second People's Hospital The Second Hospital of Liaocheng Affiliated to Shandong First Medical University Liaocheng China; ^4^ Department of Cancer Chemotherapy Zhangqiu District People's Hospital Jinan China; ^5^ Department of Oncology The Shouguang People’s Hospital Shouguang China

**Keywords:** cervical cancer, EMT, GDF15, migration, TGF‐β/Smad2/3 pathway

## Abstract

Growth differentiation factor 15 (GDF15), a member of the transforming growth factor β (TGF‐β) superfamily, is a prognostic biomarker of cervical cancer. In addition, GDF15 has been reported to enhance the migration of colorectal cancer cells and liver cancer stem‐like cells. However, the mechanism by which GDF15 promotes cervical cancer cell migration is not completely understood. Here, we report that GDF15 expression is enhanced in cervical cancer tissues, as well as in cultured cervical cancer cells. ShGDF15 transfection markedly inhibited expression of Vimentin, N‐cadherin and Snail1, and resulted in up‐regulation of E‐cadherin expression in HT‐3 and HeLa cells. Moreover, knockdown of GDF15 suppressed wound healing rate and reduced the number of invasive cells. Furthermore, knockdown of GDF15 significantly suppressed the expression of phosphorylated Smad2 and Smad3. The addition of TGF‐β1 partially abolished the inhibitory effects of GDF15 knockdown on the migration and invasion of cervical cancer cells. In summary, we report here that GDF15 knockdown inhibits migration and invasion of cervical cancer cells *in vitro* through the TGF‐β/Smad2/3/Snail1 pathway.

## Graphical abstract

The expression of growth differentiation factor 15 (GDF15) is increased in cervical cancer tissues, especially in metastatic cervical cancer tissue, as well as cultivated cells. Knockdown of GDF15 markedly affects epithelial–mesenchymal transformation‐related gene expression and suppresses the migration and invasion abilities of cervical cancer cells through the transforming growth factor‐β/Smad2/3/Snail1 pathway.

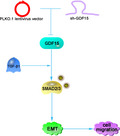

**Abbreviations**
CIS: cervical carcinoma *in situ*
EMT: epithelial–mesenchymal transformation
ERK1/2: extracellular regulated protein kinases1/2
GDF15: growth differentiation factor 15
ICC: invasive cervical carcinoma
MMP: matrix metalloproteinase
NC: normal cervical tissue
p‐: phosphorylated
PI3KAQ9/AKT: phosphatidylinositide 3‐kinases/protein kinase B
qRT‐PCR: quantitative real‐time PCR
STAT3: signal transducer and activator of transcription 3
TGF‐β: transforming growth factor β

Cervical cancer is a malignant tumor that affects the female reproductive system, and its incidence ranks the first among all female tumors in some developing countries [1]. Human papillomavirus infection is the main factor leading to cervical cancer [2]. Although advances have been achieved in the therapy of patients with cervical cancer, the prognosis for tumor metastasis of patients still needs improvement in some patients [2]. Therefore, it is important to search for the specific molecules and biomarkers for oncogenesis, diagnosis and therapy of cervical cancer.

The secreted protein growth differentiation factor 15 (GDF15) belonging to the transforming growth factor β (TGF‐β) superfamily displays the typical structural characteristics of the TGF‐β superfamily [3, 4]. Human gene GDF15 is located on chromosome 19p12‐13.1 [5]. The precursor protein of GDF15 contains 308 amino acids. After furin protease cutting, the preprotein of GDF15 becomes mature protein and secretes out of the cell. GDF15 exerts function on the secreted cell itself and surrounding cells through autocrine and paracrine [4]. In the normal human body, the expression of GDF15 shows obvious tissue specificity, mainly distributed in the prostate, kidney and pancreas [6], whereas its expression was increased at the lesions of kidney and liver and in several cancers [6, 7].

GDF15 regulates various cellular functions in cancers. For example, the high expression of GDF15 inhibited the metastasis of lung adenocarcinoma via regulation of the TGF‐β/Smad signaling pathway, and GDF15 plays a vital role in the mediation of proliferation and apoptosis of prostate cancer cells [8, 9, 10]. In addition, GDF15 activated extracellular regulated protein kinases1/2 (ERK1/2) through autocrine and paracrine to promote the development of breast cancer [11]. In colorectal cancer, GDF15 regulated the TGF‐β/Smad2/3 pathway to facilitate tumor migration [12]. A previous study pointed out that GDF15 expression was elevated in cervical cancer, and it had been suggested as a novel potential biomarker [13]. Moreover, Li *et al*. [14] found that GDF15 enhanced the proliferation of cervical cancer cells through phosphorylating AKT1 and ERK1/2 via ErbB2. However, the regulatory role of GDF15 on cervical cancer cell migration and the possible mechanisms have not been clarified.

Epithelial–mesenchymal transformation (EMT) is a transformation process in which epithelial cells turn into mesenchymal cells under physiological or pathological conditions [15]. In recent years, it has been reported that EMT is closely associated with metastasis of epithelial cell tumors [16]. EMT is a fundamental process in the tumorigenesis, which confers malignant properties of tumor cells in invasion and drug resistance through the activated TGF‐β/Smads, Wnt/β‐catenin, Phosphatidylinositide 3‐kinases/protein kinase B (PI3K/AKT) and IL‐6/Signal Transducer and Activator of Transcription 3 (STAT3) pathways [16, 17].

A good understanding of the regulatory mechanism of EMT may provide promising insights for cancer treatment. In this study, we explored the role of GDF15 on the migration of cervical cancer cells and its mechanism.

## Materials and methods

### Clinical specimen

Forty‐four cervical cancer tissues [23 cervical carcinoma *in situ* (CIS) and 21 invasive cervical carcinoma (ICC) tissues] were resected from patients who were enrolled at the Shouguang People's Hospital, and 19 normal cervical tissues (NCs) were collected from January 2014 to January 2017. All tissues were acquired by surgical resection and promptly frozen in liquid nitrogen for the following experiments. The patients had not received any radiochemotherapy before tissue collection. Informed consent was signed and obtained from all participants. This study was approved by the ethics committee of The Shouguang People's Hospital, and the study protocol and procedures conformed to the standards set by the latest revision of the Declaration of Helsinki.

### Cell culture and transfection

Immortalized human cervical epithelial cell line End1/E6E7 was bought from Shanghai Institute of Cell Biology, Chinese Academy of Sciences (Shanhai, China), and five cervical cancer cell lines (HeLa, C‐33a, SiHa, CaSki and HT‐3) were purchased from American Type Culture Collection (Rockville, MD, USA). End1/E6E7 cells were cultured in Ham’s F12 medium (HyClone, Logan, UT, USA), HeLa and CaSki cells were maintained in RPMI‐1640 (Gibco, USA), Minimum Essential Medium (Gibco, Waltham, MA, USA) was used to cultivate SiHa and C‐33a cells, and HT‐3 cells were cultivated in McCoy 5A medium (Gibco). All media were supplemented with 10% FBS (HyClone) and 1% penicillin–streptomycin, and all cell lines were maintained in an incubator containing 5% CO_2_ at 37 °C. The shGDF15 expression vector constructed and authenticated using PLKO.1 lentivirus vector in Genechem Co., Ltd (Shanghai, China) and the empty vector (sh‐NC) served as control groups. In brief, PLKO.1‐sh‐GDF15 vector was constructed according to the sequence of GDF15 and characteristics of the vector. The sequence of the forward oligo was AATTCAAAAAGCTCCAGACCTATGATGACTTCTCGAGAAGTCATCATAGGTCTGGAGC and the reverse oligo was AATTCAAAAAGCTCCAGACCTATGATGACTTCTCGAGAAGTCATCATAGGTCTGGAGC. LB agar plates containing ampicillin were applied to select the recombinant plasmids. After extraction and purification of endotoxin‐free recombinant plasmid, PLKO.1‐sh‐GDF15 vector and Lenti‐Easy packaging mix were cotransfected into 293T cells using Lipofectamine 2000 (Invitrogen, Carlsbad, CA, USA). Then, the cell supernatant was collected for concentration and purification. The titer of lentivirus was determined by the fluorescence counting method. The shGDF15 vector was transfected into HeLa and HT‐3 cells with added polybrene. Forty‐eight hours after transfection, puromycin (2 mg·mL^−1^) was added to select stable transfection. The transfection efficiency was measured using quantitative real‐time PCR (qRT‐PCR) and western blot. Stable transfected sh‐GDF15 cells were treated with TGF‐β1 for 24 h. The transfected cells were harvested for further study.

### Immunohistochemistry

Tissue samples were fixed with 10% formalin for 24 h. Then samples were dehydrated, transparent, waxed and embedded. Paraffin‐coated tissue blocks were sliced into 4‐ to 6‐μm sections. Paraffin sections were deparaffinized in xylene twice for 10 min each time and rehydrated with different concentrations EtOH (absolute ethyl alcohol, 95% EtOH, 80% EtOH) for 5 min each time. After deparaffinization and rehydration, the tissue sections were blocked with 3% H_2_O_2_ for 10 min. Antigen retrieval was performed with 0.01 m citrate buffer solution (pH 6.0) for 15 min. Then goat serum was added to tissues sections to seal the nonspecific binding site for 15 min. Tissues sections were incubated with the primary antibody against GDF15 (1 : 400; Abcam, Cambridge, MA, USA) at 4 °C overnight and rewarmed at 37 °C for 45 min in the next day. Subsequently, the sections were incubated with horseradish peroxidase‐labeled secondary antibody at room temperature for 20 min. Next, after the addition of diaminobenzidine for color development, the sections were counterstained using hematoxylin. Finally, the images were obtained under a microscope (Olympus, Tokyo, Japan).

### qRT‐PCR

Total RNAs were extracted using TRIzol reagent (Invitrogen) from the tissues and cells. cDNA was acquired using PrimeScript RT Reagent Kit (TaKaRa, Shiga, Japan) through reverse transcription. SYBR Premix Ex Taq II (TaKaRa) was applied for qRT‐PCR. The expression of GDF15 was quantified by the $2^{‐\Delta \Delta C_{t}}$ method, and β‐actin was used for normalization.

### Western blot and ELISA

Total cell proteins were extracted using RIPA lysis buffer (Beyotime, Shanghai, China) and separated by 10% SDS/PAGE. The target proteins were transferred onto the poly(vinylidene difluoride) membrane (Bio‐Rad, Hercules, CA, USA). After blocking with 5% nonfat milk, the membranes containing the proteins of interest were incubated with primary antibodies against GDF15 (1 : 1000; Abcam, Cambridge, MA, USA), Vimentin (1 : 2000; Abcam), E‐cadherin (1 : 1000; CST, Littleton, CO, USA), N‐cadherin (1 : 1000; CST), Snail1 (1 : 1000; CST), matrix metalloproteinase 2 (MMP2; 1 : 1000; CST), MMP9 (1 : 1000; CST), phosphorylated (p)‐Smad2 (1 : 2000; Abcam), Smad2 (1 : 200; Sino Biological, Beijing, China), p‐Smad3 (1 : 2000; Abcam), Smad3 (1 : 3000; Abcam) and β‐actin (1 : 5000; Santa, Dallas, TX, USA) at 4 °C overnight. Afterward, the horseradish peroxidase‐conjugated secondary antibodies were added and incubated for 2 h. The proteins were visualized using the enhanced chemiluminescence detection kit (EMD, Billarica, MA, USA). The cell culture supernatant was collected to detect GDF15 protein level using Quantikine ELISA human GDF15 immunoassay kit (Elabscience, Wuhan, China) according to the manufacturer’s manual.

### Immunofluorescent staining

Cells were fixed on the slides with 4% paraformaldehyde for 15 min. After being permeabilized with 0.5% Triton X‐100, the slides were blocked with goat serum for 30 min. Subsequently, the slide was probed with primary antibodies against E‐cadherin (1 : 200; CST) and Vimentin (1 : 1000; Abcam, USA) at 4 °C overnight. Next, the slides were incubated with the secondary antibody [donkey anti‐(rabbit IgG), goat anti‐(mouse IgG)1 : 200; Abcam, USA) for 2 h. Finally, cell nuclei were stained with DAPI, and the images were obtained on a confocal microscope (ZEISS, Dublin, Germany).

### Wound healing assay

Cells transfected with shGDF15 vector were seeded into a six‐well plate. The wounds were generated on the surface of cell monolayers using the 10‐µL micropipette tip. Then the cells were maintained in the medium containing 1% FBS for 24 h. The photographs of wounds at 0 and 24 h were acquired under a microscope (Olympus), and the migration rate was analyzed by image j software (National Institutes of Health, Bethesda, MD, USA).

### Transwell assay

Cells were seeded into the upper chamber precoated with Matrigel of a 24‐well plate (8‐μm pore size; BD, Bedford, MA, USA) supplied with 1% FBS. Six hundred milliliters of medium containing 10% FBS was added into the lower chamber. After being cultured for 48 h, the cells in the upper chamber were wiped off, and the cells invaded to the lower chamber were fixed with 4% polyformaldehyde and stained with crystal violet. The invasive cells were observed under a microscope (Olympus).

### Statistical analysis

All experiments were repeated three times. Data were presented as the mean ± standard deviation (SD). Statistical software spss 18.0 (SPSS Inc., Chicago, IL, USA) was used for statistical analysis. Student’s *t*‐test and one‐way ANOVA were conducted to analyze the statistical significance between different groups. A *P* value <0.05 was deemed as statistically significant.

## Results

### Up‐regulated GDF15 expression in cervical cancer tissues and cell lines

**Fig. 1:**
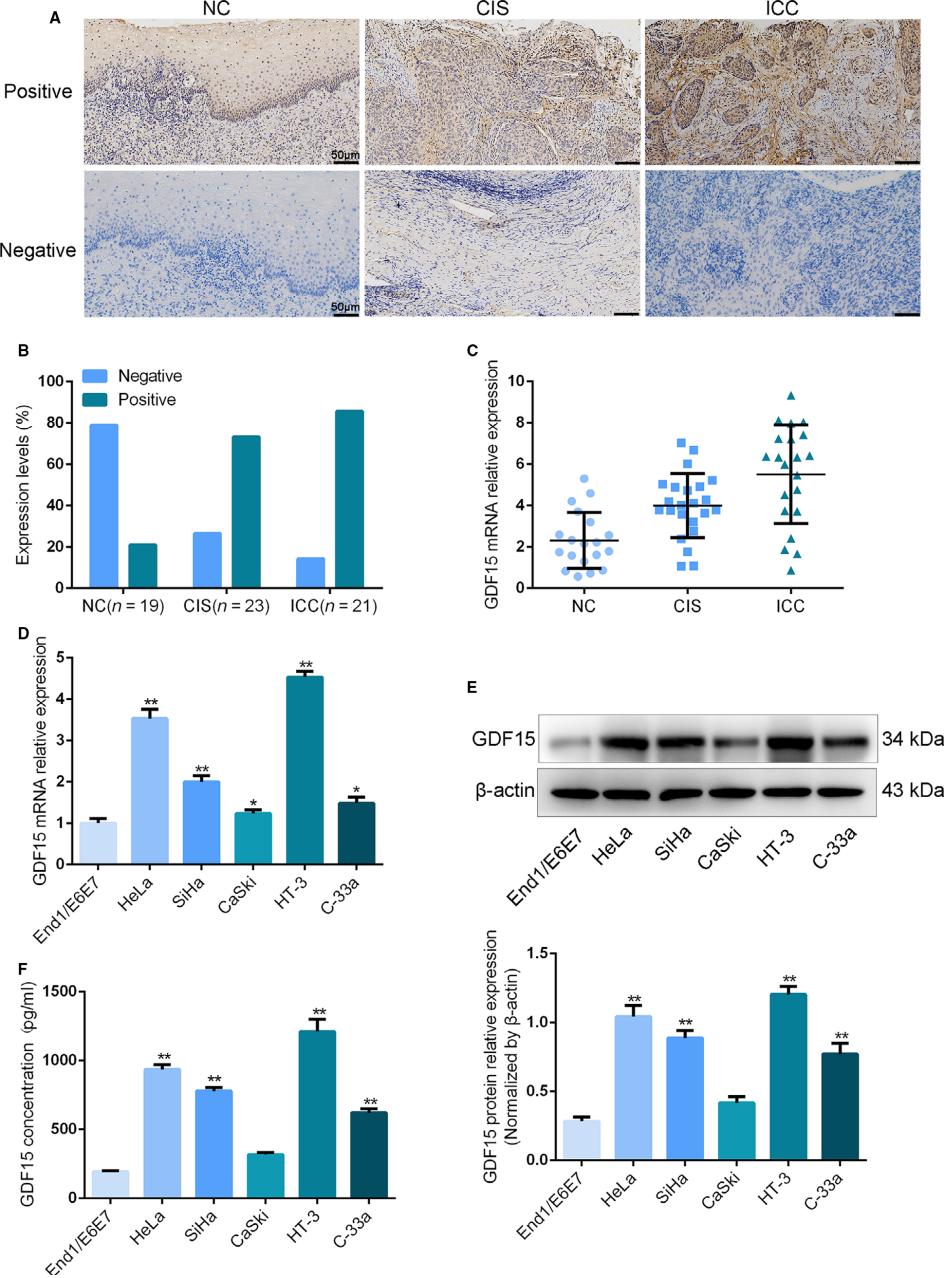
The expression of GDF15 in cervical cancer tissues and cell lines. (A) The expression of GDF15 was examined by immunohistochemistry in cervical cancer tissues. Scale bars, 50 μm. (B) The immunoreactivity scores (intensity of staining) of GDF15 analysis. (C). qRT‐PCR was performed to detect GDF15 expression in CIS and 21 ICC tissues and 19 NCs. (D–F) The mRNA (D) and protein expressions (E, F) of GDF15 were measured in cervical cancer cell lines by qRT‐PCR, western blot and ELISA, respectively. Data were presented as means ± SD for three independent experiments and were analyzed using Student’s *t*‐test. *P < 0.05, **P < 0.01.

First, we explored the expression of GDF15 in different cervical cancer tissues by immunohistochemistry assay. The results were shown in Fig. 1A. GDF15 expression was accumulated in cervical tumor tissues, especially in ICC tissues, compared with NCs. Positive GDF15 staining accounted for 21.05% in the NC group, 73.39% in the CIS group and 85.71% in the ICC group (Fig. 1B). The results of qRT‐PCR were consistent with immunohistochemistry (Fig. 1C, *P* < 0.05). Subsequently, GDF15 expression was detected in cervical cancer cell lines (HeLa, C‐33a, SiHa, CaSki and HT‐3) and human cervical epithelial cell line End1/E6E7. As illustrated in Fig. 1D,E, the mRNA and protein expressions of GDF15 were stronger in cervical cancer cell lines compared with the human cervical epithelial cell line (*P* < 0.05). The expression of GDF15 was the highest in HT‐3 and HeLa cells; thus, HT‐3 and HeLa cells were selected for the following experiments. Moreover, the result of ELISA confirmed that the level of mature GDF15 was elevated in the culture solution of cervical cancer cell lines (Fig. 1F, *P* < 0.01). Hence these results proved that GDF15 expression was augmented in cervical cancer tissues and cell lines.

### Silencing of GDF15 affected EMT‐related genes expressions in cervical cancer cells

**Fig. 2:**
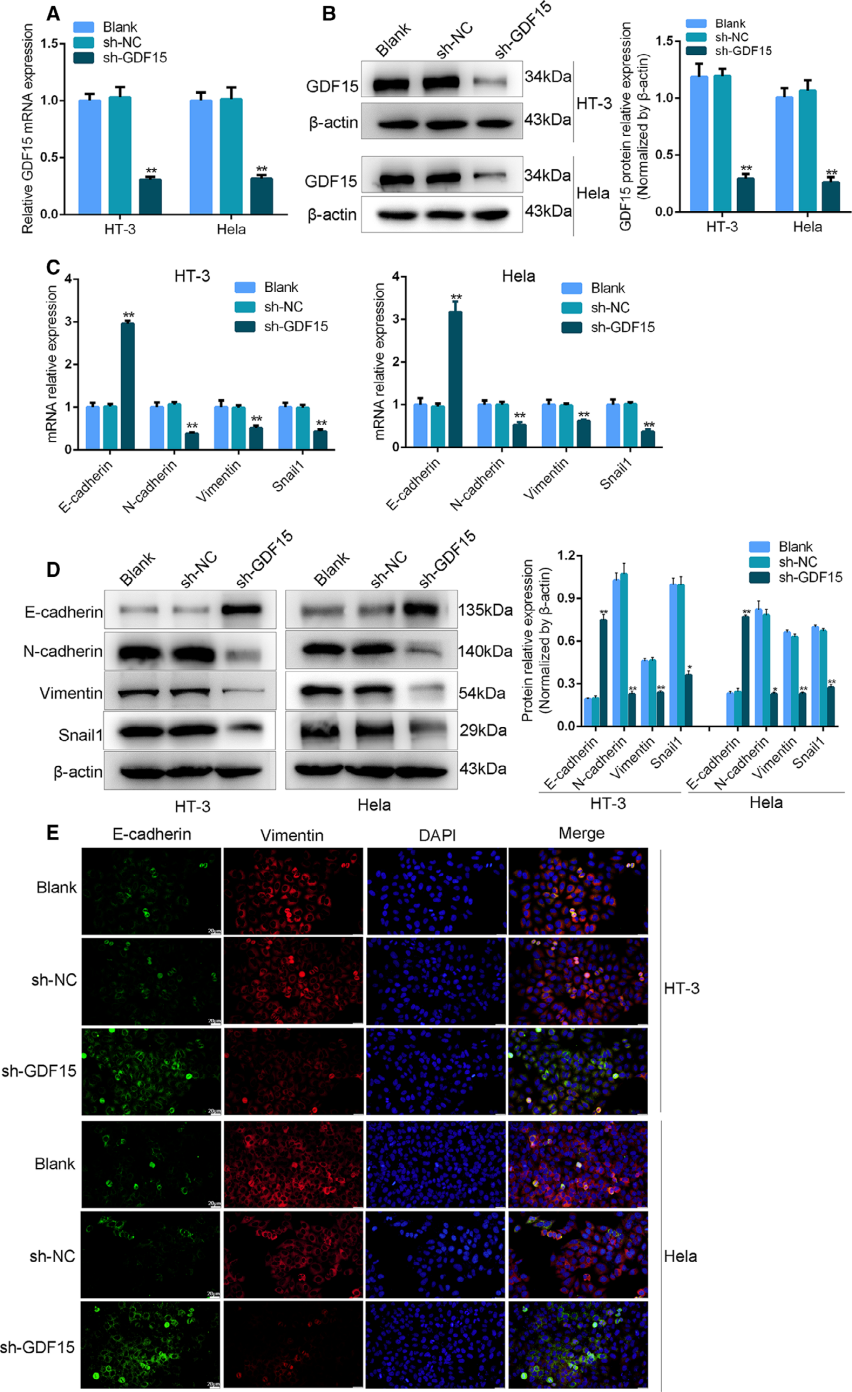
Knockdown of GDF15 inhibited the EMT‐related genes expression of cervical cancer cells. The expression of GDF15 was detected using (A) qRT‐PCR and (B) western blot assays in HT‐3 and Hela cells transfected with sh‐GDF15 or sh‐NC. (C–E) qRT‐PCR (C), western blot (D) and immunofluorescent staining (E) were applied to assess the expressions of EMT‐related genes (E‐cadherin, N‐cadherin, Vimentin and Snail1) in HT‐3 and Hela cells transfected with sh‐GDF15 or sh‐NC. Scale bars, 20 μm (*n* = 3). Data were presented as means ± SD for three independent experiments and were analyzed using Student’s *t*‐test. *P < 0.05, **P < 0.01. “Blank” indicated untransfected control.

GDF15 has been previously reported as a biomarker of cervical cancer [13], but its biological functions need to be further studied. In this section, we investigated the effect of GDF15 on EMT‐related gene expressions in cervical cancer cells. HT‐3 and HeLa cells were transfected with shGDF15 vector to knock down the endogenous GDF15. First, the outcomes of qRT‐PCR and western blot verified that the transfection of shGDF15 vector effectively reduced the expression of GDF15 in HT‐3 and HeLa cells (Fig. 2A,B, *P* < 0.01). Meanwhile, the results of qRT‐PCR indicated that knockdown of GDF15 markedly elevated the expression of epithelial cell marker E‐cadherin and down‐regulated the expressions of mesenchymal cell‐related genes (Vimentin, N‐cadherin, Snail1) in HT‐3 and HeLa cells (Fig. 2C, *P* < 0.01). The outcomes of western blot and immunofluorescence analysis also confirmed the aberrant expression of EMT‐related genes after GDF15 knockdown (Fig. 2D,E). Taken together, we found that GDF15 silencing could regulate the EMT‐related gene expressions in cervical cancer cells.

### Knockdown of GDF15 retarded the migration and invasion of cervical cancer cells

**Fig. 3:**
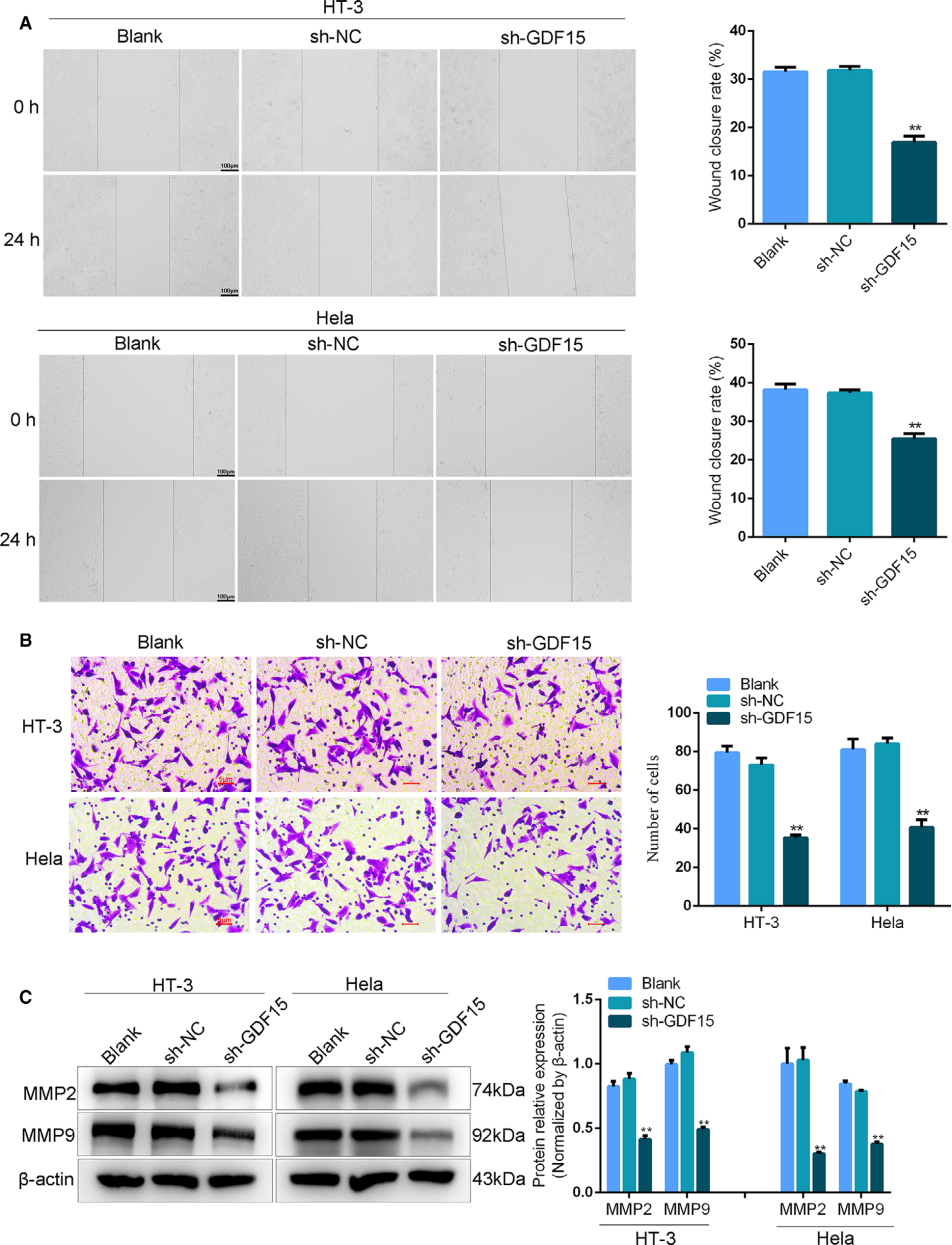
GDF15 suppression prevented the migration and invasion of cervical cancer cells. (A) Wound healing assay was performed to examine the migration ability of cervical cancer cells in HT‐3 and Hela cells transfected with sh‐GDF15 or sh‐NC. Scale bars, 100 μm (*n* = 3). (B) The invasion of GDF‐15‐suppressed HT‐3 and Hela cells was assessed by transwell assay. Scale bars, 5 μm (*n* = 3). (C) The protein expressions of MMP2 and MMP9 in HT‐3 and Hela cells transfected with sh‐GDF15 or sh‐NC were tested by western blot. Data were presented as means ± SD for three independent experiments and were analyzed using Student’s *t*‐test. **P < 0.01. “Blank” indicated untransfected control.

To investigate whether GDF15 controls the migration of cervical cancer cells through regulation of EMT‐related gene expression, we detected the invasion and migration of cervical cancer cells. Compared with the blank (untransfected control) and sh‐NC groups, the transfection of shGDF15 significantly weakened the cell migration ability in the shGDF15 group (Fig. 3A). However, there was no obvious difference in wound healing rate between the blank group and sh‐NC group (Fig. 3A). Consistently, GDF15 down‐regulation led to a dramatic reduction of the number of invasive HT‐3 and HeLa cells (Fig. 3B). Besides, MMP2 and MMP9 can degrade collagen in the basement membrane of blood vessels, which is involved in angiogenesis, tumor invasion and migration[18]. MMP9 can promote the release of activated vascular endothelial growth factor (VEGF) [19]. Therefore, we further tested the expressions of MMP2 and MMP9 in HT‐3 and HeLa cells. As shown in Fig. 3C, knockdown of GDF15 remarkedly suppressed MMP2 and MMP9 expressions. Therefore, GDF15 knockdown could impede the migration and invasion of cervical cancer cells.

### GDF15 knockdown retarded migration of cervical cancer cells through restraining the TGF‐β/Smad2/3 signaling pathway

**Fig. 4:**
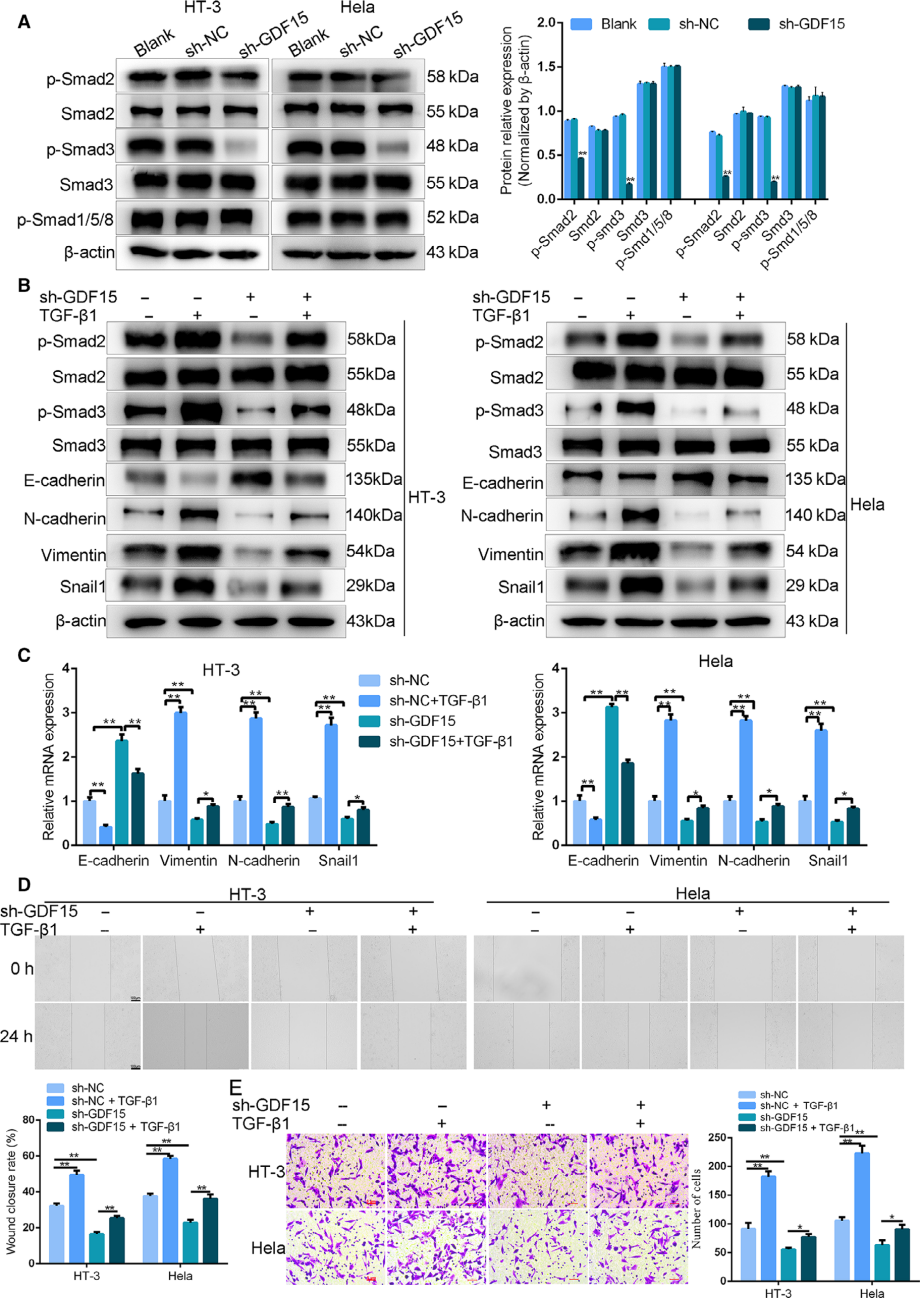
GDF15 played its roles in cervical cancer cells through the TGF‐β/Smad2/3 signaling pathway. (A) The expression levels of p‐Smad2, p‐Smad3 and p‐Smad1/5/8 were measured by western blot in HT‐3 and Hela cells, which were transfected with sh‐GDF15 or sh‐NC plasmid. (B and C) Western blot (B) and qRT‐PCR (C) were used to analyze E‐cadherin, N‐cadherin, Vimentin and Snail1 expressions in TGF‐β1‐treated HT‐3 and Hela cells, which were transfected with sh‐GDF15 or sh‐NC plasmid. The migration and invasion abilities were examined using (D) wound healing and (E) transwell assay in TGF‐β1‐treated HT‐3 and Hela cells, which were transfected with sh‐GDF15 or sh‐NC plasmid. Scale bars, 100 μm (*n* = 3) (D) and 5 μm (*n* = 3) (E). Data were presented as means ± SD for three independent experiments and were analyzed using Student’s *t*‐test and one‐way ANOVA. *P < 0.05, **P < 0.01. “Blank” indicated untransfected control.

Next, we explored the possible molecular mechanisms of how GDF15 inhibited the migration of cervical cancer cells. Because GDF15 belonged to the TGF‐β superfamily, we detected whether GDF15 affected the activity of the TGF‐β/Smad signaling pathway. The outcomes of western blot assay revealed that transfection of shGDF15 distinctly diminished p‐Smad2 and p‐Smad3 expressions but did not affect p‐Smad1/5/8 expression in HT‐3 and HeLa cells (Fig. 4A). At the same time, the expressions of Smad2 and Smad3 remained unchanged (Fig. 4A). Our finding illustrated that GDF15 regulated the TGF‐β/Smad2/3 signaling pathway in cervical cancer cells.

To further investigate whether GDF15 affected cervical cancer metastasis through mediating the TGF‐β/Smad2/3 signaling pathway, we treated HT‐3 and HeLa cells with Smad2/3 activator TGF‐β1 (10 ng·mL^−1^) after shGDF15 transfection. Interestingly, addition of TGF‐β1 increased the expressions of p‐Smad2, p‐Smad3 and EMT‐related genes (Vimentin, N‐cadherin, Snail1), counteracting the down‐regulation of p‐Smad2, p‐Smad3 and EMT‐related genes (Vimentin, N‐cadherin, Snail1), which was induced by shGDF15 knockdown in HT‐3 and HeLa cells (Fig. 4B,C). Meanwhile, TGF‐β1 down‐regulated E‐cadherin expression and reversed the promotion of E‐cadherin expression, which was initially induced by shGDF15 (Fig. 4B,C). Subsequently, the consequences of wound healing and transwell assays confirmed that TGF‐β1 partially abolished the inhibitory effect of shGDF15 on the migration and invasion of HT‐3 and HeLa cells (Fig. 4D,E). Overall, these data strongly supported that GDF15 knockdown suppressed migration and invasion of cervical cancer cells through the TGF‐β/Smad2/3 signaling pathway.

## Discussion

In this study, we verified that the expression of GDF15 at both mRNA and protein levels was significantly increased in cervical cancer tissues and cell lines. Our results were consistent with the findings of Li *et al*. [14], which proposed that GDF15 was up‐regulated and accelerated cervical cancer cell proliferation. GDF15 is highly expressed in multiple disease states, such as organ injury, hypoxia, pressure and tumor, and it promotes cell apoptosis and inhibits tumor growth [4, 6]. In contrast, the increased expression and secretion of GDF15 promote cancer development by regulating cell proliferation, immune response, angiogenesis, infiltration and metastasis [20]. For instance, overexpression of GDF15 promotes EMT and metastasis of colorectal cancer cells [12]. GDF15 is highly expressed in tumor tissues and serum of patients with glioma, which contributes to the proliferation of glioma malignancy cells [21]. Although GDF15 is regarded as one of the best biomarkers for the diagnosis of malignant tumors [22], its biological roles in tumors need to be further studied. A previous study suggested that GDF15 was a novel biomarker for the prognosis of cervical cancer [13]. In the present research, we further investigated the influences of GDF15 on the migration of cervical cancer cells. First, we transfected shGDF15 vector into HT‐3 and HeLa cells to knock down the endogenous GDF15. qRT‐PCR and western blot analysis revealed that shGDF15 successfully down‐regulated the expression of GDF15 in HT‐3 and HeLa cells. Moreover, shGDF15 transfection hindered the migration and invasion of HT‐3 and HeLa cells. This was consistent with the effect of GDF15 on the migration of osteosarcoma and pancreatic cancer cells [23, 24]. MMP2 is involved in the degradation of tumor basement membrane and extracellular matrix [18], and the complex formed by MMP2 and MMP14 promotes the formation of blood vessels [25]. In live cancer, TPX2 inhibited the expression of MMP2 and MMP9 to suppress cell invasion and metastasis [26]. In this study, the results of western blot showed that knockdown of GDF15 weakened the expression of MMP2 and MMP9. These results supported that GDF15 played an oncogenic role in cervical cancer.

Accumulating evidences suggest that the TGF‐β/Smads signaling pathway plays a crucial role in mediating the EMT process. The TGF‐β/Smads signaling pathway was first found to induce EMT in mouse breast epithelial cells and later was proved to take part in the tumor EMT process [27, 28]. In tumor cells, the TGF‐β/Smads signaling pathway is associated with the invasion and metastasis of tumor cells induced by EMT through regulating the downstream transcription factors, such as Snail and α‐SMA [29]. Previous research has shown that GDF15 was involved in the migration of colon cancer and lung adenocarcinoma by regulating the TGF‐β/Smads signaling pathway [8, 12]. In this study, we explored the regulatory role of GDF15 on the TGF‐β/Smads signaling pathway in cervical cancer cells. Knockdown of GDF15 reduced the expressions of p‐Smad2 and p‐Smad3, whereas it showed no effect on p‐Smad1/5/8 expression, which suggested that GDF15 regulated the Smad2/3 pathway in cervical cancer cells. Furthermore, we explored whether GDF15 modulated cervical cancer cell migration through mediating the TGF‐β/Smad2/3 signaling pathway. The result showed that the addition of TGF‐β1 elevated the expressions of Vimentin, N‐cadherin and Snail1 and inhibited E‐cadherin expression. At the same time, exogenous TGF‐β1 facilitated the migration of cervical cancer cells. More importantly, TGF‐β1 partially reduced the regulatory effect of GDF15 silencing on EMT‐related genes expressions and the migration of cervical cancer cells. Based on the earlier data, we concluded that GDF15 silencing could suppress the migration of cervical cancer cells via regulating the TGF‐β/Smad2/3/Snail1 pathway.

In conclusion, our study showed that silencing of GDF15 significantly inhibits the progression of cervical cancer through the TGF‐β/Smad2/3/Snail1 pathway. Our findings may provide new insight into the treatment of cervical cancer.

## Conflict of interest

The authors declare no conflict of interest.

## Author contributions

LL and RZ performed the majority of the experiments and wrote the manuscript. HY and DZ collected data and performed the experiments. J Liu and J Li analyzed data. BG designed and modified the manuscript. All authors agreed to submit the manuscript.

## Data Availability

The analyzed datasets generated during this study are available from the corresponding author on reasonable request.
